# Identifying Player Types to Tailor Game-Based Learning Design to Learners: Cross-sectional Survey using Q Methodology

**DOI:** 10.2196/30464

**Published:** 2022-04-04

**Authors:** A E J Van Gaalen, J Schönrock-Adema, R J Renken, A D C Jaarsma, J R Georgiadis

**Affiliations:** 1 Anatomy & Medical Physiology Section Department of Biomedical Sciences of Cells and Systems University Medical Center Groningen, University Groningen Groningen Netherlands; 2 Wenckebach Institute for Education and Training University Medical Center Groningen Groningen Netherlands; 3 Cognitive Neuroscience Center Department of Biomedical Sciences of Cells and Systems University Medical Center Groningen, University Groningen Groningen Netherlands; 4 Faculty of Veterinary Medicine Utrecht University Utrecht Netherlands

**Keywords:** gamification, serious games, game-based learning, medical education, computers, new technology, focus group, play, qualitative, player types, taxonomy, theory, framework

## Abstract

**Background:**

Game-based learning appears to be a promising instructional method because of its engaging properties and positive effects on motivation and learning. There are numerous options to design game-based learning; however, there is little data-informed knowledge to guide the choice of the most effective game-based learning design for a given educational context. The effectiveness of game-based learning appears to be dependent on the degree to which players like the game. Hence, individual differences in game preferences should be taken into account when selecting a specific game-based learning design.

**Objective:**

We aimed to identify patterns in students’ perceptions of play and games—player types and their most important characteristics.

**Methods:**

We used Q methodology to identify patterns in opinions on game preferences. We recruited undergraduate medical and dental students to participate in our study and asked participants to sort and rank 49 statements on game preferences. These statements were derived from a prior focus group study and literature on game preferences. We used by-person factor analysis and varimax rotation to identify common viewpoints. Both factors and participants’ comments were used to interpret and describe patterns in game preferences.

**Results:**

From participants’ (n=102) responses, we identified 5 distinct patterns in game preferences: the social achiever, the explorer, the socializer, the competitor, and the troll. These patterns revolved around 2 salient themes: sociability and achievement. The 5 patterns differed regarding cheating, playing alone, story-telling, and the complexity of winning.

**Conclusions:**

The patterns were clearly interpretable, distinct, and showed that medical and dental students ranged widely in how they perceive play. Such patterns may suggest that it is important to take students’ game preferences into account when designing game-based learning and demonstrate that not every game-based learning-strategy fits all students. To the best of our knowledge, this study is the first to use a scientifically sound approach to identify player types. This can help future researchers and educators select effective game-based learning game elements purposefully and in a student-centered way.

## Introduction

In health professional education, there has been a growing interest in game-based learning because of its engaging properties and positive effects on students’ motivation and learning [1]. Yet, the understanding of how and when to implement game-based learning in educating health professionals remains in its infancy [1] as well as in other educational domains [2]. Although there are myriad ways to design game-based learning strategies [2-4], there is little good-quality evidence to guide the choice of the most effective game-based learning design in a given educational context [1]. This, in turn, may increase the likelihood of choosing suboptimal or even counterproductive game-based learning strategies [5]. Hence, there is a need for empirical research to inform future game-based learning design [1].

Some scholars have stated that educational games are designed by academics who do not understand the culture, art, and science of games [6-8]. This may result in educational learning tools that can either be a success or a failure with respect to playability and engagement. On the other hand, games developed by game designers with little or no understanding of the theory and practice of game-based learning can be fun to play but are also hit-or-miss with respect to educational goals and outcomes. Indeed, designing an educationally sound game-based learning tool is a challenging task and depends highly on the synergy between pedagogy and engagement [5-8].

Practical applications of game-based learning have not been substantiated by a significant body of scientific research [1,3,9], which could be interpreted as corroboration for the abovementioned assertions. Researchers in health professional education generally take an educational approach to game-based learning without considering the body of knowledge available in the field of game research. For example, most game-based learning research in health professional education focused on one specific game attribute (ie, the effects of scoring and rewards) [1,2,10], although many other game attributes have also been investigated [3,11]. Moreover, game elements that motivate some learners may actually demotivate others [12-14] indicating that personal preference is a crucial element for motivation to play [15-18]. Game [19] and game-based learning [5,20] research consistently demonstrated that people vary greatly in what they like in play and games. Outside the domain of education for health professionals, individual differences in age, gender, culture, and personality play a role in a person’s preferences for specific types of play, games, and responses to different game-based learning designs [20]. Linking personality traits with game-based learning design solutions that best fit each particular trait has been shown to improve learner experience (eg, perceived playfulness) [12,21-27], motivation [28-31], and performance [28,30]. Hence, preferences should be considered in designing game-based learning strategies to engage and motivate an entire cohort of students (not only a subgroup).

**Figure 1:**
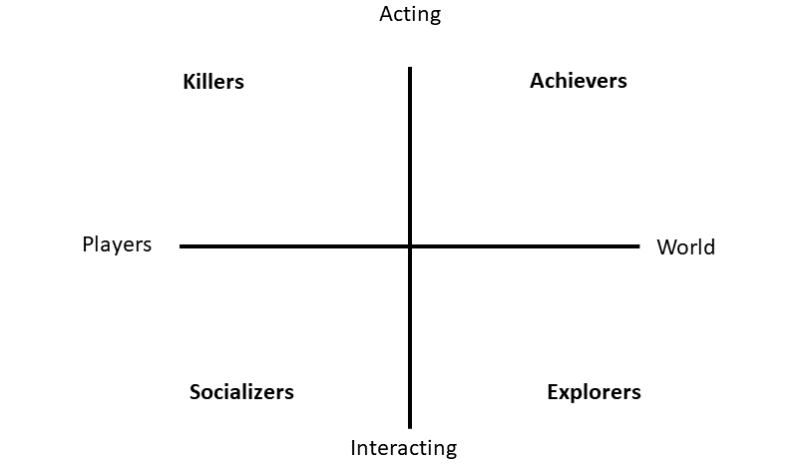
Typology [18] with 4 player types based on 2 themes: player vs world and acting vs interacting.

In the field of game research, the concept of player types is used to characterize users who share preference for a specific type of play, which enables game designers to create an optimal user experience [32]. In an earlier and probably best-known player typology [18], users of a multiplayer role-playing game were classified on the basis of two in-game behaviors—(1) acting versus interacting and (2) world versus player—which resulted in 4 different player types: socializers (users who like to interact with other players, eg, the game is a tool to meet other people), explorers (users who like to interact with the world, eg, discover new areas, and immerse themselves in the game world), achievers (users who like to act on the world, eg, prefer gaining rewards, points, and equipment from the game world), and killers (users who like to act upon other players, eg, thrive on beating other people) (Figure 1). Since then, various player types have been proposed [17,19,33-35]. However, there are major concerns with these player typologies. Many are not supported by empirical evidence [35]. Instead, they are based on researchers’ prior experience in developing games [18,33], on nonscientific literature [36,37], or on combinations of some of the aforementioned player types [38]. Player typologies based on empirical data [17,19,39] tend to be based on research into a specific game genre, which means the typologies may be biased and incomplete. In addition, surveys that were used (eg, Likert-scale surveys [17,19,39]) may have unnecessarily limited respondents’ answers and, thereby, researchers’ interpretations. Hence, important information may have been overlooked [40].

In this study, we aimed to identify player types among a representative group of education for health professional students, independent of game context. As the first study of its kind, we aimed to explore the widest possible range of preferences for game and play in this group. We formulated the following research question: What patterns in students’ perceptions of play and games (ie, player types) can be identified and what are their most important characteristics?

## Methods

### Design

To investigate students’ perceptions of play, we applied Q-methodology [41], which is a mixed methods research technique that aims to account for all key subjective viewpoints on a certain matter [42]. The qualitative component of Q-methodology allows the expression of subjective opinions to be considered, and the quantitative component uses statistical analysis in order to group participants with shared opinions. Q-methodology is used to cluster individuals based on shared opinions rather than based on latent variables, which is the case in regular factor analysis (or R-methodology). The Q-methodology technique has been used before in medical education, although for different purposes (eg, for identifying different patterns of self-regulating learning behavior [43-45]). Q-methodology is characterized by two main stages: (1) designing a set of statements and let participants sort that set of statements, and (2) by-person correlation and factor analysis of a sample of the included participants.

### Ethics

We obtained ethical approval for this study from the Netherlands Association for Medical Education (NVMO 2019.1.11).

### Setting and Participants

This study was conducted in May 2019 at the Faculty of Medical Sciences of the University of Groningen, the Netherlands. The 6-year undergraduate medical and the 6-year undergraduate dental curriculum both consist of a 3-year Bachelor and a 3-year Master’s phase. In both curricula, teachers used face-to-face and web-based teaching methods and sometimes apply game-based learning, but not on a regular or structural basis.

We invited all medical and dental students (3000 eligible undergraduate students) to participate in our study by posting an announcement on the web-based learning environment (called Nestor) of the university. Participants were informed about the purpose and procedures of the study. Their participation was anonymous, voluntary, and confidential. Participants had the right to withdraw from the study at any time. All participants provided informed consent. In appreciation for their time and effort, each participant received a 5€ (approximately US $5.66) gift certificate.

Although there is no decisive minimum or maximum number of participants for performing Q-methodology research [43], generally, the number of statements should exceed the number of participants [46], and 40 to 60 participants is considered adequate [47,48]. To achieve a highly diverse sample—which is recommended for Q-sort analysis [46,48]—we purposively selected participants. Therefore, we asked participants to complete a sociodemographic questionnaire (eg, age, gender, and whether or not they considered themselves a gamer) prior to the sorting process that also included a question about the participant’s favorite game. For our purposive sample, we included only participants who had specified a favorite game. We identified the game genre to ensure each game genre was represented evenly in the final sample. In addition, only participants who had performed the sorting task in 12 minutes or more were included. We conducted pilot testing and found that the average sorting time was 25 minutes (range 2-3 minutes) and that reading the statements and swiftly sorting the statements took at least 12 minutes; thus, for less than 12 minutes, the sort was regarded as ill-considered. If there were participants with identical favorite games, only one participant was included. Participants’ preferences for modality (digital or analog) had to be distributed as evenly as possible across game genres; the male-to-female ratio had to be evenly distributed across game genres; and medical and dental student had to be evenly distributed across game genres. If a decision about inclusion or exclusion of a participant could not be made based the preceding criteria, the decision was made by rolling dice.

### Statement Set

There is no single correct way to compile a set of statements in Q-methodology [41]. In general, the Q sample size is 40 to 80 statements [41,48], and the number of statements should exceed the number of participants [46]. A set containing too many statements can make the sorting process an exhausting and burdensome task, whereas a set containing too few statements may result in inadequate coverage of the topic of game preferences [41]. By sorting and prioritizing each statement from the statement set, individual participants provided us with a model of their view on their own game preferences. Statements should be carefully selected since their nature limits what can be expressed by a participant [49].

We aimed to develop a set of statements in which each statement was unique and made its own original contribution, and all statements together covered the full range of game preferences. Statements were based on the findings of an earlier focus group study [5] among medical and dental students (n=58) with no experience in game-based learning but widely varying experiences in play and games that had been conducted to obtain perspectives on leisure time and academic education. To make sure that the statement set covered as many aspects of game preferences as possible, we also examined player type studies [17-19,50] that possibly addressed different game preferences. This resulted in an initial set of 136 statements. We grouped the statements into 28 themes, duplicates were removed, and statements were translated into English and reworded to start with the phrase “I like games that….” in order to improve clarity and make sorting more intuitive for participants [41]. The final set (Table 1) consisted of 49 statements and was piloted by 3 medical students. Based on their feedback, we considered the final statement set to meet our abovementioned aims.

**Table 1:** Statement set and factor array.

Statement	Factors				
	1	2	3	4	5
1. I like games in which people help each other.	1	0	−2	3	−1
2. I like to see how others learn a new game.	−2	0	−1	−3	−3
3. I like games with easy wins.	−3	−3	−1	2	−2
4. I like games which create an atmosphere of sociability.	3	0	−3	1	1
5. I like games that let me build relationships.	2	0	−3	1	0
6. I like games that let me play in teams.	2	−1	0	2	0
7. I like to play games to maintain relationships.	1	−3	−2	2	0
8. I like games that let me play on my own.	−3	3	3	0	−1
9. I like games in which I can create something.	1	1	0	2	2
10. I like games that allow different ways of winning.	2	3	1	2	−2
11. I like games that use luck to enhance my odds of winning.	−1	−3	−1	−1	−4
12. I like games with a good storyline.	1	4	1	3	1
13. I like games in which I can influence the storyline.	0	4	0	0	−1
14. I like games in which I need to actively participate.	3	1	0	4	1
15. I like games in which I know the other players.	2	0	−2	0	3
16. I like games in which I can solve a difficult part / puzzle.	1	2	3	4	3
17. I like to improve my gameplay by searching for new techniques.	0	2	0	−2	−3
18. I like games in which I learn new things (eg, knowledge/skills)	3	2	3	1	0
19. I like games in which I can act differently than I usually do in real life.	−2	1	−2	0	0
20. I like games which make you feel immersed in your own world.	−2	3	−1	−1	4
21. I like games that let me apply a strategy.	4	2	2	0	1
22. I like games in which I can bluff.	−1	−1	−2	0	3
23. I like games that have trading elements.	0	−2	−1	1	2
24. I like games in which I can negotiate.	1	1	−1	0	0
25. I like games that can be played differently than they are intended.	−1	1	−3	−2	2
26. I like games in which I can cheat.	−4	−2	−3	−4	3
27. I like games in which other players cheat.	−4	−4	−4	−4	−3
28. I like games in which I can be fanatic.	3	−2	1	−1	1
29. I like games in which I can play strictly by the rules.	0	−3	1	2	−2
30. I like games to which I can bring modifications.	−2	1	0	−2	0
31. I like games in which I can obtain as many points as possible.	−1	0	2	0	1
32. I prefer losing with lots of rewards over winning with very few.	−3	−1	−1	−1	−2
33. I like games which show me my progression.	2	2	2	1	0
34. I like games which let me have items that others don’t manage to collect.	−2	−2	0	−1	0
35. I like games which have a reward at stake.	0	1	0	−1	−1
36. I like games in which I can get my revenge after losing.	0	−2	1	−1	1
37. I like games that show everyone that I’ve won.	−1	−2	1	−3	−3
38. I like games in which I can prove to the other players that I am the best.	−1	−1	2	−2	−2
39. I like games that use competition as a way to improve myself.	2	0	3	−1	−2
40. I like games in which I can annoy other players.	−1	−4	−2	−3	2
41. I like games that use competition to defeat other players.	1	0	1	−3	1
42. I like to be the best in a game.	1	−1	4	−2	2
43. I’m a good loser.	0	−1	−4	0	−4
44. Winning is important to me.	−1	−1	4	−2	2
45. I like games in which I can play alone against a game or computer	−3	3	2	1	−1
46. I like games which let me stay anonymous.	−2	1	1	1	−1
47. I like to get better in a game.	4	2	2	3	3
48. I like games that use a lot of different materials (eg**,** dices, cards, fake money)	0	0	0	1	−1
49. I like games in which losing is okay.	0	−1	−1	3	−1

### Sorting Procedure

Participants performed the sorting procedure using a web app (Q-sorTouch), in which the 49 statements were randomly presented. Participants were asked to drag and drop each statement into 3 piles: agree, neutral, disagree. After sorting all the statements, they had to refine their 3 piles by ranking the statements into a Q-sort grid ranging from −4, extremely disagree, to +4, extremely agree. In Q-methodology, the number of statements that can be assigned to each scale point are fixed and represent a quasi-normal distribution (Table 2) [41]; thus, participants placed the 2 statements with which they disagreed most under −4 and the 2 with which they agreed most under +4.

**Table 2:** Quasi-normal distribution.

Position	−4	−3	−2	−1	0	+1	+2	+3	+4
Number of items	2	4	6	8	9	8	6	4	2

The sorting procedure ended when all statements were placed in the fixed distribution and the participants felt that the final sort represented their viewpoint. In the final stage of the data collection, participants provided answers to open-ended questions to elaborate on the rationale behind their sort (eg, why statements were assigned to the extreme ends).

### Statistical Analysis

To identify groups of participants with shared, but distinct, viewpoints (ie, who subjectively ranked the 49 statements in a similar way), we conducted by-person factor analysis using dedicated software (PQMethod, version 2.35; developer: J Atkinson), which we later verified with formulas [48] in MATLAB (version R2020a; The MathWorks).

Because each sort was correlated with every other sort, the correlation matrix of the participants’ sorted statement sets (ie, *sorts*) was used to identify factors (ie, groups of respondents whose Q-sorts were statistically similar) by subjecting the correlation matrix to varimax rotation [41]. Varimax rotation generates a factor solution according to the best mathematical solution (while maintaining an orthogonal basis) [48]. Only factors with eigenvalues >1 and on which at least 2 participants are loaded significantly (*P*<.01) were accepted [41,48,49], which corresponded to a factor loading >0.37, calculated using 2.58 × (1 / √ (number of items in the Q set) [41,48]. Since our aim was to extract patterns that were unique, participants loading on more than one factor were not used for the construction of a factor. This is in line with the procedures applied in other Q-methodology studies [41,43,44,48].

A range of factor solutions were generated. To describe patterns of the participants’ game preferences, each factor solution was interpreted in conjunction with qualitative data from participants’ responses in the final stage of the sort. To facilitate factor interpretation, ideal Q-sorts were computed for each factor. These so-called factor arrays are weighted averages of sorts loading on that factor [41,49]. A group of 9 independent researchers individually interpreted all factor solutions and were asked to identify the solution with the highest number of viewpoints while providing distinct and clearly interpretable factors.

## Results

### Overview

A total of 102 students volunteered to participate in our study and completed the sorting procedure. On the basis of their statements about their favorite games, we identified 7 game genres: action games (n=7), adventure games (n=6), party games (n=13), simulations or sports games (n=15), strategy games (n=35), puzzle games (n=14), and role-playing games (n=10). Consequently, we excluded 60 participants: 2 participants did not provide their favorite game; 10 participants performed the sort in less than 12 minutes; 36 participants had duplicate favorite games (eg, 11 participants stated the game *Settlers of Catan*); 9 participants (4 favorite digital games, 2 females and 3 male students) to ensure a more even distribution of these variables; and 3 participants, by the roll of the dice. The sample consisted of 42 participants (dental students: n=13; medical students: n=29) having 41 different favorite games, of whom 31 were female and 11 were male, with a mean age of 23.3 years (SD 4.0; range 18-42). Of the 42 participants, 15 participants considered themselves to be gamers. Nine sorts were confounded, and 3 sorts did not load significantly on any of the factors (factor loading <0.37; Table 3).

**Table 3:** Factor matrix.

Q sort	Factor				
	1	2	3	4	5
1	0.383	0.675^a^	0.211	0.182	0.170
2	0.559^a^	0.096	0.089	0.282	0.166
3	−0.041	0.670^a^	0.109	0.284	−0.019
4^b^	0.521	0.522	0.294	0.132	0.312
5	0.578^a^	0.183	0.147	0.031	0.025
6	0.226	0.753^a^	−0.057	0.188	0.048
7	−0.176	0.124	0.584^a^	0.171	0.109
8^c^	0.168	0.364	0.087	0.336	−0.094
9^c^	0.303	−0.296	0.316	0.361	−0.255
10	0.423^a^	0.288	−0.089	0.354	−0.065
11^b^	0.208	0.100	0.521	0.414	−0.334
12	0.501^a^	0.104	0.143	0.160	0.176
13^b^	0.531	0.253	0.535	0.241	−0.045
14	0.344	0.054	0.014	0.526^a^	0.060
15	0.368	0.562^a^	−0.065	0.387	0.110
16^b^	0.402	0.645	0.178	−0.003	0.106
17	−0.113	0.358	0.135	0.639^a^	−0.131
18	0.357	0.166	−0.137	−0.035	0.569^a^
19	0.191	0.703^a^	0.134	0.134	−0.392
20^b^	0.607	0.214	0.073	0.621	0.094
21	0.047	0.686^a^	0.188	−0.011	0.292
22	0.550^a^	0.097	0.038	0.328	−0.293
23	−0.011	0.695^a^	0.308	−0.158	0.055
24	0.708^a^	−0.067	0.237	0.159	0.009
25	0.341	0.175	−0.323	0.556^a^	−0.092
26	0.068	0.380	0.721^a^	0.222	0.131
27^b^	0.658	0.162	−0.202	0.448	−0.175
28	−0.041	0.089	0.246	−0.025	0.598^a^
29	0.320	0.019	0.052	0.479^a^	0.257
30	0.391	0.046	0.276	0.572^a^	−0.045
31^b^	−0.324	0.504	0.439	0.133	0.057
32	0.709^a^	−0.288	0.065	0.265	0.099
33	0.357	0.118	0.658^a^	−0.274	−0.137
34	0.551^a^	0.058	−0.100	0.042	−0.137
35	0.512^a^	0.226	0.223	0.195	−0.064
36	0.508^a^	0.128	−0.086	0.129	0.210
37	0.138	0.064	0.625^a^	−0.030	0.071
38^c^	0.173	0.096	0.190	0.294	−0.299
39^b^	0.199	0.435	0.043	0.073	0.452
40	0.474^a^	0.108	0.055	0.026	0.041
41^b^	0.467	0.327	0.580	−0.106	−0.030
42	0.596^a^	0.272	0.272	0.233	−0.070

^a^A defining sort for a specific factor.

^b^A confounded Qsort (multiple loadings).

^c^A Q-sort with a factor loading <0.37.

### Factor Interpretation

#### Overview

Solutions with up to 5 factors were obtained. The 5-factor solution was retained after analysis by 9 independent researchers because it represented 5 clearly distinguishable patterns in students’ perceptions of play and games and had the highest percentage agreement between researchers (88.9%).

Of the 42 included sorts, 30 loaded significantly (factor loading >0.37; Table 3) on 1 of the 5 factors. These patterns are presented below, with sociodemographic information about the participants and a relevant statement to illustrate each pattern. For example, in pattern 1, statement 21 is in the *extremely agree* position (21: +4) in that factor array (Table 1). To give a concise (but oversimplified) overview of the patterns, we chose a descriptor for each that reflected its interpretation in a broad sense.

#### Social Achiever

Pattern 1 comprised 12 participants with significant factor loadings (female: 9, male: 3; age: mean 23.7 years, range 18-42 years), of whom 7 were medical students, and 5 were dental students. Of the 12 participants, 5 self-identified as gamers. Favorite game genres were strategy (n=5), action (n=3), party (n=2), and simulation or sport games (n=2). Preferred modality was distributed evenly; 6 participants favored analog games, and 6 participants favored digital games.

Participants in Pattern 1 shared the opinion that playing is a social act (4: +3; 5: +3), playing alone or in an individual competition with the self was, therefore, generally disliked (8: −3; 45: −3).

What I really like in games is to collaboratively achieve something meaningful.

Student 3

The act of social togetherness was not enough for these participants, as they also expressed the need to obtain something meaningful through play (18: +3). Participants loading on this pattern tended to work hard and fanatically toward that goal (28: +3; 3: −3).

Notably, strategy was liked to a great extent (21: +4), which seemed attributable to the fun of being able to play socially and achieving something together (18: +3). Student 67 mentioned,

In my opinion, games are way more fun when you play them with friends …. besides, they will give us way longer fun when it is possible to apply a strategy…. This keeps the game interesting and fun for a longer time.

#### Explorer

Pattern 2 comprised 7 participants with significant factor loadings (female: 5, male: 2; age: mean 23.1 years, range 20-31 years), of whom 6 were medical students, and 1 was a dental student. Of the 7 participants, 5 participants self-identified as gamers. Favorite games genres were adventure (n=3), role-playing (n=2), action (n=1), and puzzle games (n=1). The majority (n=5) favored a digital modality over an analog modality.

Pattern 2 was characterized by a need for immersion (20: +3), which was especially satisfied through story-driven games (12: +4, 13: +4). Student 21 stated,

A good game must drag me into the story and not let go until I am finished.

These participants generally liked games that granted them substantial autonomy (10: +3; 29: −3) to explore and alter the game (25: +1; 30: +1). They seemed to be drawn to exploring the potential of the game rather than searching for sociability in play (8: +3; 45: +3; 7: −3). Participant 12 stated,

For me, gaming is something that I can do primarily on my own.

These participants played for their own sake or individual pleasure. (6: −1; 7: −3).

#### Competitor

Pattern 3 comprised 4 participants with significant factor loadings (female: 2, male: 1; age: mean 22.2 years, range 21-23 years). Two participants were medical, and 2 participants were dental students. Two participants self-identified as gamers. Favorite game genres in this group were puzzle (n=2) and simulation or sports games (n=2). Of the 4 participants, 3 students favored a digital modality over an analog modality.

Hunger for competition was the defining aspect of pattern 3 (39: +3)—not only winning or being the best (42: +4; 44: +4), but also parading their supremacy was considered important compared to the other patterns (37: +1; 38: +2). As stated by Student 93,

I am very competitive, I want to win every game and I want to show that to everyone.

Losing was therefore greatly disliked (43: −4). These participants shared the opinion with those described by pattern 2 that other game players were not important to them and they would rather play alone (8: +3); however, whereas participants described by pattern 2 had neither a strong preference nor a dislike for social togetherness as a characteristic of play (4: 0; 5: 0), participants described by pattern 3 found sociability in play unnecessary (4: −3; 5: −3; 7: −2; 15: −2). As Student 23 stated,

I play for myself, not for others.

Thus, *competitors* like competition that does not involve collaboration with others but is directed against other players (since they want to prove they are the best (37: +1; 38: +2)) or a nonplayable character. Student 23 stated,

I like to play independently of other players but with an opponent; so, against a computerized opponent.

#### Socializer

Pattern 4 comprised 5 participants with significant factor loadings (female: 4, male: 1; mean age 26.2 years; range 2-9 years), of whom 4 were medical students, and 1 was a dental student. Of the 5 participants, 2 participants identified themselves as gamers. Favorite game genres in this group were party (n=2), role-playing (n=2), and action games (n=1). The majority favored an analog modality (n=4) over a digital modality (n=1).

Participants described by Pattern 4 and Pattern 1 had similar characteristics. They valued collaborative play (5: +1; 6: +2); however, whereas being fanatic was important in Pattern 1, in pattern 4, participants did not have the urge to focus on winning (44: −2) or being fanatic (28: −1). They generally disliked competition (41: −3; 42: −2; 44: −2).

Winning is not important to me, I just enjoy working together with others and having a good time together.

Student 68

This concept of “having a good time” seemed to be a recurrent characteristic for Pattern 4. Games were seen as a means for social togetherness (7: +2; 1: +3) that should depend on nothing but sociability. Losing should be okay (49: +3) and winning should be easy (3: +2); however, participants felt that active participation would be needed to have a good time (14: +4).

### Troll

Pattern 5 comprised 2 participants with significant factor loadings (female: 2; age: mean 23.5 years, range 2-5 years), and both were both medical students. One student self-identified as a gamer. One student favored action games, the other student favored simulation or sports games. Both students favored a digital modality (n=2).

Having the ability to exploit game mechanics to cheat (26: +3), annoy other players (40: +3), and bluff (22: +3) was important for these 2 participants compared with participants described by the other patterns. Such behavior seemed to be the result of boredom or laziness and not really being interested in the game itself. Notably, these participants were not inclined to invest time to learn new techniques (17: −3) but, paradoxically, wanted to get better in a game (47: +3), did not like to see others learn the game (2: −3), and were inclined to play games differently than intended when the game would take too much time (25: +2; 29: −2).

I like it when a game requires little prior knowledge. It is much simpler and easier to play.

Student 51

**Figure 2:**
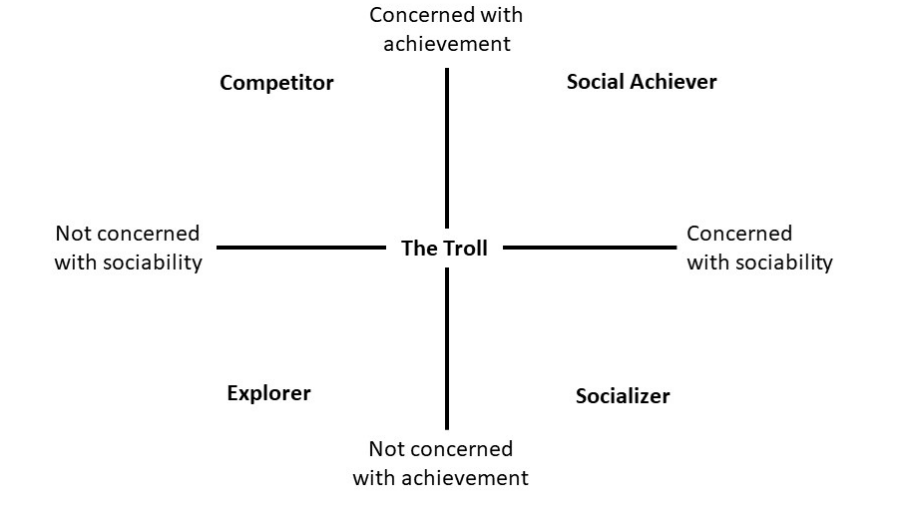
Theoretical framework illustrating player types in relation to sociability and achievement themes.

Figure 2 presents a theoretical framework illustrating different player types in relation to sociability and achievement themes.

## Discussion

### Principal Findings

We aimed to improve the understanding of game-based learning design, in general, and of game-based learning in health professional education, in particular. We contended that player typology, a concept that has been used to inform game design and game play, may be relevant to game-based learning design. To the best of our knowledge, this study is the first to investigate player types in a student cohort, outside of a game design context, using a methodology deliberately aimed to accommodate the largest variety in game preferences. We found that, in a cohort of medical and dental students with likely similar academic interests and intellectual ability, there was considerable variability in play preferences. We identified 5 distinct and clearly interpretable patterns in game preferences, which can be considered player types: the social achiever, the explorer, the socializer, the competitor, and the troll. Given that only a few game elements are applied in education for health professionals research—predominantly points and rewards [1]—our findings indicate that there is room for improvement within game-based learning design; the current variety of game-based learning designs in education for health professionals seems too limited to be able to tailor game-based learning to students’ game preferences to improve learning through motivation and engagement.

Each player type has distinct characteristics. *Social achiever*s like to play collaboratively to achieve something meaningful. They like competition and difficult games, preferably in teams. In contrast, *explorers* are drawn to the game’s story and immersive elements of play rather than winning and team play. *Competitors* on the other hand, thrive by winning and competition and would rather not depend on others. *Socializers* (much like *social achievers*) play for sociability, and interaction with other players is important to them. Yet, whereas competition is important to *social achievers*, *socializers* would rather play to find a sense of sociability and togetherness. They look for easy wins just to have a good time. *Trolls* like games in which they can annoy other players, bluff, and cheat.

Two themes (Figure 2) are salient in the player types that we identified, and likewise in scientific and grey literature on play and player typologies [17,19,35,39,51-56]: sociability and achievement. *Competitors* and *social achievers* like the achievement aspect of play, however, *explorers* and *socializers* instead preferred playing for the storyline or to enjoy playing together, respectively. *Social achievers* and *socializers* are driven by sociability, collaboration, and interaction; *explorers* and *competitors*, however, seem less prone to these traits or only need others to prove their supremacy. *The troll* is more ambivalent about sociability and achievement aspects of games than other player types and is, therefore, situated at the intersection of achievement and sociability.

Interestingly, the main themes identified in our theoretical framework bear similarity to the very first and often-cited player typology [18], which was based on a sample with homogeneity in terms of the preferred game, instead of homogeneity in terms of academic interest and which lacked any empirical basis. The fact that our study (which followed a more rigorous scientific approach) resulted in a similar typology may suggest that the existence of player types in a cohort is stable. Our scientific approach led to the identification of the *explorer* and the *socializer*, player types that have also previously been described [18]. We identified the *social achiever*, a player type that seems comparable with that of “achiever [18].” We additionally identified 2 other player type—*the competitor* and *the troll*—whereas in [18] only one other player type, namely the “killer [18],” which, upon close inspection, includes troll-like aspects (eg, annoying other players) as a social component (acting on other players). In our study, these characteristics appeared in other player types. We identified *the troll* and *the competitor* as separate player types. The achiever [18], with its social component was therefore interpreted as *social achiever*. The reason for these differences between both typologies may be that we also included games involving teamplay as a play genre in our framework, since we aimed to avoid selection bias from using only one or a few specific game genres to identify player types. The earlier typology [18] did not include teamplay, probably because it was based on a multiuser dungeon game that included role playing, player versus player, and chat functions but rarely team effort. We also found differences in relation to the dimensions on which the player types varied. Whereas the dimensions *world versus player* and *action versus interaction* have been previously described [18] our empirical evidence supported *achievement* and *sociability* as player type dimensions. As a result, *competitors* and *socializers* were opposites in our framework (instead of “killers” and “explorers” [18]).

The *troll* as a player type has not been identified in previous studies [20]. Remarkably, however, the troll phenomenon is well known in the field of problematic gaming and internet use [57,58]. *Trolling* is defined as deliberately trying to create distress or conflict via provocation, for instance, for the purpose of deception or disruption [58]. More than one-third of American millennials said they engaged in the act of trolling [59] and an immensely popular digital game, called Among Us, is based on the concept of trolling (ie, sabotaging and causing chaos [60]). This suggests that the game-related behavior of trolling is not rare or marginal. Although the relevance of this player type to game-based learning design is unclear, this player type might also be pertinent outside the field of education for health professionals.

### Strength and Limitations

The player types in this study represent a broad spectrum of views on games and play. One of the strengths of this study is that the comprehensive set of statements was derived from prior research among medical and dental students [5] and supplemented with statements taken from existing player type studies. Furthermore, a solid scientific method was used to account for all key subjective viewpoints on game preferences, and we included of a variety of participants (independent from game context) to prevent selection bias on game genre. In addition, we discussed multiple factor solutions, sought advice from expert authors [41], and verified Q-methodology software results. In doing so, we added a new perspective to literature on player types and game-based learning by identifying 5 patterns that were distinct, characteristic, and could be considered player types.

This study had some limitations: (1) In the interpretation of our patterns, we cannot (and do not) claim to be exhaustive with respect to all viewpoints on game preferences in the entire population. While Q-methodology is a method that aims to capture variety and heterogeneity, our participant group was relatively homogenous (medical and dental students). Therefore, we cannot claim that replication of our study in a different educational context would yield the same outcomes. However, by adding statements from prior (nonmedical) studies on player types in the statement set, and by using stratification to provide profuse and varied participants’ opinions, we feel that the quantitative aspect of the Q-methodology (ie, analyzing participants’ rankings using multivariate data reduction techniques) helped us detect meaningful patterns and connections in game preferences. This, in turn, may provide future researchers with a starting point to investigate the generalizability of our results. (2) In a recent study [5], we showed that game elements are possibly context dependent (ie, aspects that motivate play may not necessarily play a motivating role in game-based learning). For instance, although competition was liked and named trivial in play in leisure time, students considered it stressful and unwanted in play focused on learning. Since we did not ask participants to keep a specific learning environment in mind when they answered the question about their game preferences, their answers may not reflect their game-based learning preferences. (3) We aimed to reduce selection bias by selecting participants independent of game context, however, we do not know whether they had a specific game or context in mind when they performed the sorting procedure. (4) We chose to adopt the 5-factor solution after rigorous discussions and with the help of 3 independent researchers. Although this allowed us to detect a new player type (the *troll*), few students had significant factor loadings on this player type. Nevertheless, this player type adhered to the widely accepted rules for including a factor in Q-methodology and helped explain the largest variety in play preferences [58].

By using Q-methodology, we aimed to explain as much variety in existing game preferences as possible; thus, our player types are extreme ends of a spectrum on game preferences. The factor arrays that construct these player types are the combined average of all sort loadings on that player type. Therefore, there is very little chance that a participant’s sort will load 100% on a specific player type and fully match its definition [41]. Indeed, all sorts demonstrated characteristics of all player types, and no sort loaded 100% on one player type. Yet, most sorts loaded clearly on one player type.

### Practical Implications and Future Research

Systematic reviews indicate that, often, game-based learning strategies are selected based on researchers’ personal opinions rather than theory or a conceptual framework [1,2,61]. Additionally, there is a tendency in game-based learning strategies to use scoring and reward, especially in gamification [1,61]. Our taxonomy provides a novel theoretical framework that may help to tailor game-based learning strategies to student preferences. Future research is needed to investigate whether such tailoring would result in increased effectiveness of applying game-based learning in education.

Based on our findings, all player types except *explorers* might need the presence or participation of other players to be optimally motivated to continue playing. To develop game-based learning strategies that optimally engage and motivate the majority of students, multiplayer options appear to be critical. However, this feature is currently overlooked in game-based learning strategies in current practice [1,11,61].

Our theoretical framework and corresponding factor arrays indicate that preferences for multiplayer modalities can be diverse and are not limited to sociability [62], social media [63], a chat function [34], and message boards [64]. *Competitors*, for instance, need other players or computerized opponents to triumph over and show their supremacy, *social achievers* need other players to work with, *trolls* need other players to annoy, and *socializers* need other players to have a good time together. By including each player type in a game-based learning-strategy, the complex and dynamic interaction between player types can turn game-based learning into a meaningful strategy for every student. For example, although *trolls* might only make a small contribution to the overall player population, their actions can have major impact on social play and interaction [65-68], much more than, for example, the actions of *social achievers*. The inclusion of *trolls* in game-based learning design can unite socially oriented players by giving them a common foe. Future research should explore how each player type can contribute to multiplayer game-based learning strategies to enhance collaborative learning.

Future research can focus on investigating whether the range of opinions on play vary significantly across students as a function of the academic level or discipline they are enrolled in, for instance, a medical or a nonmedical group, or medical specialization. Such findings would provide an understanding for future student-specific game-based learning designs. Game preferences might be dependent on context [5] or the players' current needs [31]. For instance, in the playground game called Tag, one player is *it* and chases the other players in an attempt to tag them by touching them. Then the tagged player becomes *it* and starts chasing the others to tag someone else. This means that, when being *it*, a player must adopt the *competitor* player type (ie, competing and winning from the others), while the others (who are getting chased) can adopt the *social achiever* or even *troll* player type to act as a group against the one that is *it*. Likewise, other digital games (eg, Among Us) perhaps also use changing player types, where one is sometimes a troll and, at other times, needs to take on the role of the social achiever [60]. This raises the question whether player types are in search of a specific game design or does the game design elicit different types of behavioral responses (ie, player types). This might also suggest that game designers should adhere to the entire diversity of player types to ensure inclusion of all participants of the game-based learning strategy.

As a first step in this direction, we aimed to investigate the prevalence of player types among medical and dental students. This may not only provide more evidence for the existence of the currently identified typology in education for health professional students, it may also shed light on the true diversity of player types within medical and dental education. Furthermore, it may improve our understanding of whether the current educational strategy focusing on the achievement-oriented player type is effective and can be justified or whether it might be better to tailor game-based learning strategies to individual player types.

### Conclusion

We identified 5 clear and distinct patterns of game preferences. These patterns represent player types that differ in terms of the player type dimensions *achievement* and *sociability*. Our taxonomy and accompanying factor arrays can be used to tailor game-based learning design to students’ game preferences to optimize game-based learning effectiveness.
